# Changes in Psychiatric Symptoms in Swedish Mothers Who Took Part in Project Support: An Intervention for Mothers Exposed to Intimate Partner Violence With Children Who Have Developed Conduct Problems

**DOI:** 10.1177/10778012231203622

**Published:** 2023-09-26

**Authors:** Helena Draxler, Fredrik Hjärthag, Maria Tillfors, Kjerstin Almqvist

**Affiliations:** 1 590934 Department of Social and Psychological Studies, Karlstad University, Karlstad, Sweden

**Keywords:** intimate partner violence, intervention, social support, posttraumatic symptoms, psychiatric symptoms

## Abstract

Project Support (PS) is an evidence-based individual support and parenting program developed for mothers exposed to intimate partner violence (IPV) whose children have developed conduct disorders. This Swedish feasibility study focuses on changes in the mothers’ psychiatric symptoms, in relation to social and emotional support received as part of PS. In a within-subject design in a naturalistic setting (i.e., 10 social service units), mothers (*n* = 35) reported a significant decrease in symptoms, but from an individual perspective, most mothers still suffered from clinical levels of psychiatric symptoms. The need for additional interventions for mothers exposed to IPV is discussed.

## Introduction

Regardless of family structure, all types of intimate partner violence (IPV) negatively affect both the victim and the children in the family (Brown, 2008; Campbell, 2002; Devries et al., 2013; Vu et al., 2016). Globally and throughout history, mothers have always been in the majority regarding exposure to IPV, and despite variance in prevalence, about 30% of all mothers aged 15 or more years have been exposed to physical and/or sexual IPV during their lifetime (Devries et al., 2013). This is a global social problem that needs to be addressed (Moulding et al., 2021). Mothers exposed to IPV have been shown to be at high risk of suffering from both physical injuries and psychiatric symptoms, such as pain, emotional distress, suicidal thoughts, and, in particular, depressive and posttraumatic stress symptoms (Bacchus et al., 2018; Ellsberg et al., 2008; Pill et al., 2017). Few general interventions addressing psychiatric symptoms take IPV into account, and therefore, aspects such as safety and risks are not included in treatment (Pill et al., 2017). Furthermore, since mothers exposed to IPV are commonly of childbearing age, children will likely also be exposed and at risk of developing psychiatric symptoms (Fong et al., 2019). Accordingly, interventions for mothers and children exposed to IPV are often structured both to address mothers’ mental health and to improve their parenting capacity so they can meet their children's needs (Austin et al., 2019; Chiesa et al., 2018; Howell et al., 2015). Project Support (PS) is an intervention with such a structure.

### Project Support (PS)

The abusive partner or caregiver seldom takes responsibility for the damage done to either the mother or the child, leaving the mother alone to bear total responsibility for herself and her child's health and development. In such a vulnerable situation, the woman often needs help and support. PS is an intervention for mothers exposed to IPV and who often alone need to care for their children (aged 3–9 years) who also have developed adjustment problems due to the subjected violence. The intervention targets the abused mother and comprises two main components: social and emotional support and parenting skills training. The purpose of the social and emotional support is to enhance the mental well-being of the mother during and after staying in a domestic violence shelter. The support includes safety planning, psychoeducation, problem-solving, validation, and financial and/or legal help. The parenting skills training is intended to help the mother repair and care for her child's development in the aftermath of IPV. Therefore, the training focuses on maternal sensitivity, warmth, and functioning with the aim of decreasing behavioral disorders in the child. The average length of treatment is about 20 sessions over 8 months (Jouriles et al., 2009).

PS has been evaluated in repeated randomized controlled trials (RCTs) and follow-up evaluations in the USA, showing useful results in terms of improving parenting capacity and decreasing children's behavioral problems (Jouriles et al., 2001, 2009, 2010; McDonald et al., 2006, 2011). The demonstrated effects of the intervention qualified PS to be chosen for a feasibility study in Sweden, when effective methods for children exposed to parental IPV were requested by the National Board of Health and Welfare (Broberg et al., 2011). A feasibility study is intended to build knowledge of whether the effects of a method are sustainable in a new context and/or whether adaptation is needed (Bowen et al., 2009). Swedish feasibility studies of PS have indicated that its positive effects in terms of improved parenting capacity and decreased behavioral symptoms in children were sustainable, but that adjustments concerning parenting skills were needed to better fit the Swedish cultural context (Draxler et al., 2019, 2020). However, the effects of PS on IPV-exposed mothers’ psychiatric symptoms have been less investigated and are less clear in both the US and Swedish contexts (Draxler et al., 2019; Jouriles et al., 2001, 2009, 2010; McDonald et al., 2006, 2011). A previous RCT study in the USA found no significant difference in terms of mothers’ psychiatric symptom improvements between PS and comparison groups after intervention, with trauma symptoms and global psychiatric symptoms decreasing in both conditions (Jouriles et al., 2009). Given the nonspecific group-level results in the USA and a need for further knowledge of feasibility in the new Swedish context, any evaluation of the social and emotional support component of PS should explore three key aspects: first, whether the social and emotional support are associated with decreased psychiatric symptoms in mothers exposed to IPV; second, how each mother changes (i.e., improves or worsens) and how this change is connected to her symptom levels (i.e., cut-off limits for psychiatric illness) and exposure to IPV; and third, aspects of PS transferability concerning circumstances for giving and receiving social and emotional support when implementing the method in a new context (e.g., Swedish Social Services).

The general aim of this study was to explore the relationship between psychiatric symptoms, IPV exposure, and received social and emotional support in Swedish mothers taking part in the PS intervention. The specific research questions were as follows:

1. Do mothers who have received the PS intervention display decreased global psychiatric symptoms and posttraumatic stress symptoms?
2. Is there a relationship between mothers’ global psychiatric symptoms, posttraumatic stress symptoms, IPV exposure, and received PS social and emotional support?

## Method

### Procedure

This study, a within-subject design without a control group, was conducted in a Swedish naturalistic setting with a convenience sample. Social service agencies in eight municipalities and two domestic violence shelters (i.e., 10 units in total) participated. Agencies volunteered to participate when information about the study was disseminated by researchers in a network concerning children exposed to IPV. The units financed their own participation and were responsible for the legislative, regulatory, and insurance coverage of participating caregivers and their children.

In Sweden, the social services are obliged to offer support to women, men, and their children who have been exposed to IPV. The offered interventions are free of charge. To investigate the needs of caregivers and children seeking support, a regular assessment conducted by a social worker precedes the decision for assistance. If the caregiver and child meet the inclusion criteria, which include (a) the caregiver having previously been exposed to IPV (but not currently) and (b) they are seeking help for a child with psychological symptoms, the caregiver is informed about the study and asked for consent. Most of the caregivers had had previous regular contact with social services but expressed that the support had been insufficient.

The screening process for participating in PS, whether for research or to receive regular service from the social service, did not differ. Accordingly, before the caregivers participated in an intervention (i.e., PS), their needs and situations were customarily assessed based on the routines of each respective social service agency. The assessment and decision-making processes were not transparent to the researchers, and each social service unit had its own routine on which the decisions were based.

Participants were informed that they could end their participation in the study at any time without stating a reason and that under no circumstances would this affect their right to receive social service support. In the study, measures of exposure to violence, global psychiatric symptoms, and posttraumatic stress symptoms in the mothers were assessed pretreatment (T1) and posttreatment (T2). The study was approved by the Regional Ethical Review Board (no. 2013/115) and was conducted in accordance with the Declaration of Helsinki (World Medical Association, 2013). The processes of recruitment for and participation in the research lasted from August 2013 to December 2016, as described elsewhere (Draxler et al., 2019).

### Participants

A total of 34 biological mothers and one father (all exposed to IPV) volunteered for the study and participated in the intervention. In the following, the sample will be referred to as “mothers,” and this choice of nomenclature has its pros and cons. The concept of “parents” or “caregivers” could have been an alternative, but IPV is, in reality, gendered, with most victims being women. Using the concept of “parent” risks hiding this aspect.

All mothers spoke Swedish, and most of the mothers (*n* = 23) were born in Sweden. Regarding the rest, three were born in Europe, seven outside Europe, and two of unknown origin. A total of 26 mothers were single and had separated from the abusive partner and three lived with a new partner. Six were still married but did not necessarily live with the formerly abusive partner, and the violence had, according to the mothers, stopped. Eleven mothers were employed, 13 relied on various forms of public financial assistance, and 11 had unknown financial situations. The sample was representative of caregivers seeking protection in domestic shelters or caregivers that were assisted by social services in their homes in terms of gender, origin, and occupation.

Of the 27 attending counselors (three men and 24 women), most (94%) had a university degree, and over 80% had been in their profession for over 5 years. Ten counselors worked in pairs during the home visits, but most worked individually. Most counselors administered PS to one mother only; however, one counselor worked with three mothers and five counselors with two mothers each. The counselor mainly responsible for administering the treatment to each mother completed a fidelity and evaluation form for each session. All counselors were trained and supervised by the originator of the method. The treatment has been described in detail elsewhere (Draxler et al., 2019).

### Attrition

Of the 35 participants, 25 (24 mothers and one father) completed the postintervention assessment. Three mothers, all living in domestic violence shelters, did not meet the inclusion criterion for the analysis, which was participating in at least six PS sessions. This criterion was based on the number of sessions said to be needed to produce a demonstrable psychological treatment effect (Nielsen et al., 2016). Seven mothers could not be reached at the time of the posttreatment assessment for various reasons: they were, for example, hiding from their former partners, had moved without providing a new address, or were in mental health care. Another mother did not complete the trauma symptom questionnaire (Impact of Event Scale–Revised (IES-R)) (*n = *24).

There were no significant differences between the dropouts and the remaining sample concerning trauma symptoms (IES-R, *p = .*460), global psychiatric symptoms (Hopkins Symptom Checklist-25 (HSCL-25), *p =* .918), or exposure to IPV (CTS2, *p =* .288) at the first assessment (T1) before the intervention.

Twenty-five mothers completed the postassessment form; for two of them, however, the fidelity forms were missing because the associated logbooks had unfortunately not been submitted by the counselors.

### Measures

The mothers completed three instruments concerning themselves: the Conflict Tactic Scale 2 (CTS2) was used at T1 to capture their exposure to IPV, and two questionnaires were used to assess psychiatric symptoms (HSCL-25 and IES-R before treatment (T1) and after the final PS session (T2). Counselors completed a fidelity and evaluation form for each session.

### Measuring Exposure to IPV

CTS2 and its 39 items were used to determine the extent to which *psychological aggression*, *physical assault*, *sexual coercion*, and *injury* were inflicted on the mothers during the past year and over their lifetimes (Straus et al., 1996). The response choices were the numbers of times the violent acts had occurred, scored using midpoint values according to the procedure outlined by Straus et al. (1996). Previous research indicates that CTS2 has adequate reliability, with subscales ranging from α = .79 to α = .95 (Straus et al., 1996). Cronbach's α for CTS2 at T1 was .92 for the total score.

### Measuring Psychiatric Symptoms

HSCL-25 is a short form of Symptom Checklist-90 (SCL90) that measures mental health and the degree of global psychiatric symptoms. HSCL-25 comprises 25 items rated on four-point Likert scales ranging from 1 (“not at all”) to 4 (“extremely”). In this study, the General Severity Index (GSI) was used, which indicates the level of distress, with the total score being divided by the number of items (Derogatis et al., 1974). HSCL-25 has adequate internal consistency, which for the total score is α = .94 (Glaesmer et al., 2014). Cronbach's α for HSCL-25 at T1 was .95.

IES-R measures traumatic symptoms related to a specific traumatic event, using the subscales intrusion, avoidance, arousal, and total score (with arousal excluded from the last subscale). The total score without aspects of arousal was used in an attempt to capture the clinical essence of posttraumatic stress, as the arousal items screen for symptoms that are more general and could be unrelated to a traumatic event (Creamer et al., 2003). IES-R comprises 22 items scored from 0 (“not at all”) to 4 (“extremely”). The internal consistency of the IES-R total score is α = .96 (Creamer et al., 2003). Cronbach's α for IES-R total at T1 was .95.

### Measuring Given Social and Emotional Support and Treatment Satisfaction

The fidelity form used by the counselors measured treatment regularity, the extent of offered social and emotional support to the mother, the mother's receptiveness to the intervention, and the counselor's satisfaction with her/his own work in the session.

The amount of time used for social and emotional support were registered as 1 if 0–25% (i.e., a small focus), 2 if 26–75% (i.e., a medium focus, as supported by the PS manual), and 3 if 76–100% (a major focus) of the session time were used for social and emotional support to the mother. To estimate the amount of given support (compared with the time used for parenting training), the ratings (i.e., 1–3) from all sessions were summed and divided by the number of sessions. It was possible for counselors to record whether deviations from the method had occurred (e.g., total session spent on emotional support for the mother or whether the child was present and no emotional support was given to the mother). For about half of the mothers, the percentage registrations for social and emotional support were estimated by the first author using the counselors’ registrations and notes. For the other half of the mothers, the registrations were complete and counselors were specifically asked what proportion of time, in percent, in each session had been used for social and emotional support of the mother.

The frequency was calculated by using the total number of sessions divided by the total number of months from the first to the last PS session.

For each session, the counselor answered two questions: “How satisfied are you regarding your own effort as a counselor?” and “How receptive do you think the mother was to the intervention during the session?” with the responses ranging from 1 (“not at all”) to 10 (“totally”). To estimate counselor satisfaction and maternal receptiveness, the ratings (i.e., 1–10) of all sessions were summed, and the total sum was divided by the number of sessions.

### Data Analyses

To measure the efficacy of the intervention, focusing on the mothers’ psychiatric symptoms, a Wilcoxon two-tailed signed-rank test was used to measure the pretreatment (T1) to posttreatment (T2) changes in completers at the group level. Effect sizes were calculated using Cohen's *d*, with 0.2 indicating a small effect, 0.5 a medium effect, and 0.8 a large effect (Cohen, 1988). The Reliable Change Index (RCI; Jacobson & Truax, 1991) was used to categorize change at the individual level. The results of the pre- and posttreatment assessments of each participant and the test–retest reliability of HSCL-25 and IES-R were used for RCI calculations; at *z *> 1.96, the change experienced by a participant was considered statistically significant, following the Jacobson and Truax model (1991). For HSCL-25, the mean test–retest reliability of 0.84 was used (Friborg et al., 2003; Lee et al., 2008; Mouanoutoua & Brown, 1995), and for IES-R, the test–retest reliability of 0.77 was used (Eid et al., 2009). Depending on the mother's prescores on HSCL-25 and IES-R, their results were divided into two categories: above the cut-off limit, indicating a clinical level (i.e., dysfunctional), and below the cut-off limit (i.e., functional). Regarding individual differences, the posttreatment test showed whether the mother remained within clinical range after treatment according to the measures of posttraumatic stress symptoms (IES-R) and global psychiatric symptoms (HSCL-25). The term “recovered” was used when a mother had changed and no longer reached a clinical cut-off level, and the term “deteriorated” was used when she had changed and worsened.

#### Missing Data

Individual mean substitution was done for a total of 22 items missing completely at random, i.e., missing items were apportioned to 14 participants. This method is applicable when a few items are missing in clinical psychology research (Roth, 1994; Shrive et al., 2006).

A Mann–Whitney *U* test was used to measure differences between mothers who had reached symptom levels above the cut-off limit and mothers who were under the cut-off limit for both global psychiatric symptoms (HSCL-25) and posttraumatic stress symptoms (IES-R). The cut-off points, i.e., ≥1.75 for HSCL-25 and GSI and >30 for IES-R, indicate a probable need for psychological treatment (Glaesmer et al., 2014; Nettelbladt et al., 1993). By using the limits, the sample was divided into two groups, i.e., above and below the cut-off limit, which were compared regarding exposure to IPV and treatment extent/configuration focusing on social and emotional support.

When calculating the correlations, the results of the first assessment (T1) were used in calculating a Spearman correlation (two-tailed) to assess (a) the association between maternal mental health and IPV exposure and (b) the associations between IPV exposure, psychiatric symptoms, treatment frequency, and amount of time, focusing on social and emotional support.

## Results

### IPV Exposure Among Mothers Participating in PS

All mothers (*n* = 35) had some lifetime exposure to psychological violence (CTS2), and those (*n* = 25) who had been exposed during the past year reported a mean of 61.5 psychologically abusive acts (standard deviation (SD) = 60.8). Most mothers (88%, *n* = 31) had at some point in their lives been victims of physical violence, and those (*n* = 13) who had been exposed during the past year reported a mean of 20.8 physically abusive acts (SD = 52.3). Over half of the mothers (55%, *n* = 19) had at some point in life been injured as a result of IPV, and those (*n* = 13) who had been injured during the past year due to IPV had been injured a mean of 8.6 times (SD = 22.3). Mothers (*n* = 13) who had been exposed to sexual coercion during the past year reported a mean of 21.9 abusive acts (SD = 8.1), and two-thirds of the studied mothers reported some lifetime exposure to sexual IPV.

### Psychiatric Symptoms in Mothers Before and After the PS Intervention

#### Global Psychiatric Symptom Assessed Using HSCL-25

Mothers reported a pre- to postintervention change in psychiatric symptoms, although the effect size was small (0.38) (Table 1). The sample had a GSI level of 1.60 preintervention and of 1.27 postintervention.

**Table 1.** Mothers’ Psychiatric Symptoms Measured with HSCL-25 and IES-R, Pre- and Posttreatment; Means, Medians, Standard Deviations, Numbers of Participants per Outcome Variable, Levels of Significance, and Effect Sizes Posttreatment.

	Pretreatment			Posttreatment					
Instrument	*M*	Mdn	SD	*M*	Mdn	SD	*n*	*Z*	*d*
HSCL-25									
Total sum	40.05	40.00	24.39	31.69	33.00	19.98	25	−1.965*	0.38
IES-R									
Intrusion	13.92	12.50	9.22	11.89	12.00	7.87	24	−1.187	0.24
Avoidance	13.25	14.50	8.53	10.21	10.50	7.24	24	−2.301*	0.39
Arousal	10.04	10.50	7.18	8.24	7.50	5.49	24	−1.514	0.28
Total sum without arousal	27.17	25.00	16.57	22.09	21.00	14.51	24	−1.774^#^	0.33
Total sum	37.21	35.00	22.79	30.33	29.00	19.53	24	−1.915^#^	0.33

*Note.* HSCL-25 = Hopkins Symptom Checklist-25 (Derogatis et al., 1974); IES-R = Impact of Event Scale–Revised (Creamer et al., 2003).

**Significant at .01 level (two-tailed).

*Significant at .05 level (two-tailed).

# Significant at .10 level (two-tailed).

**Figure 1.**
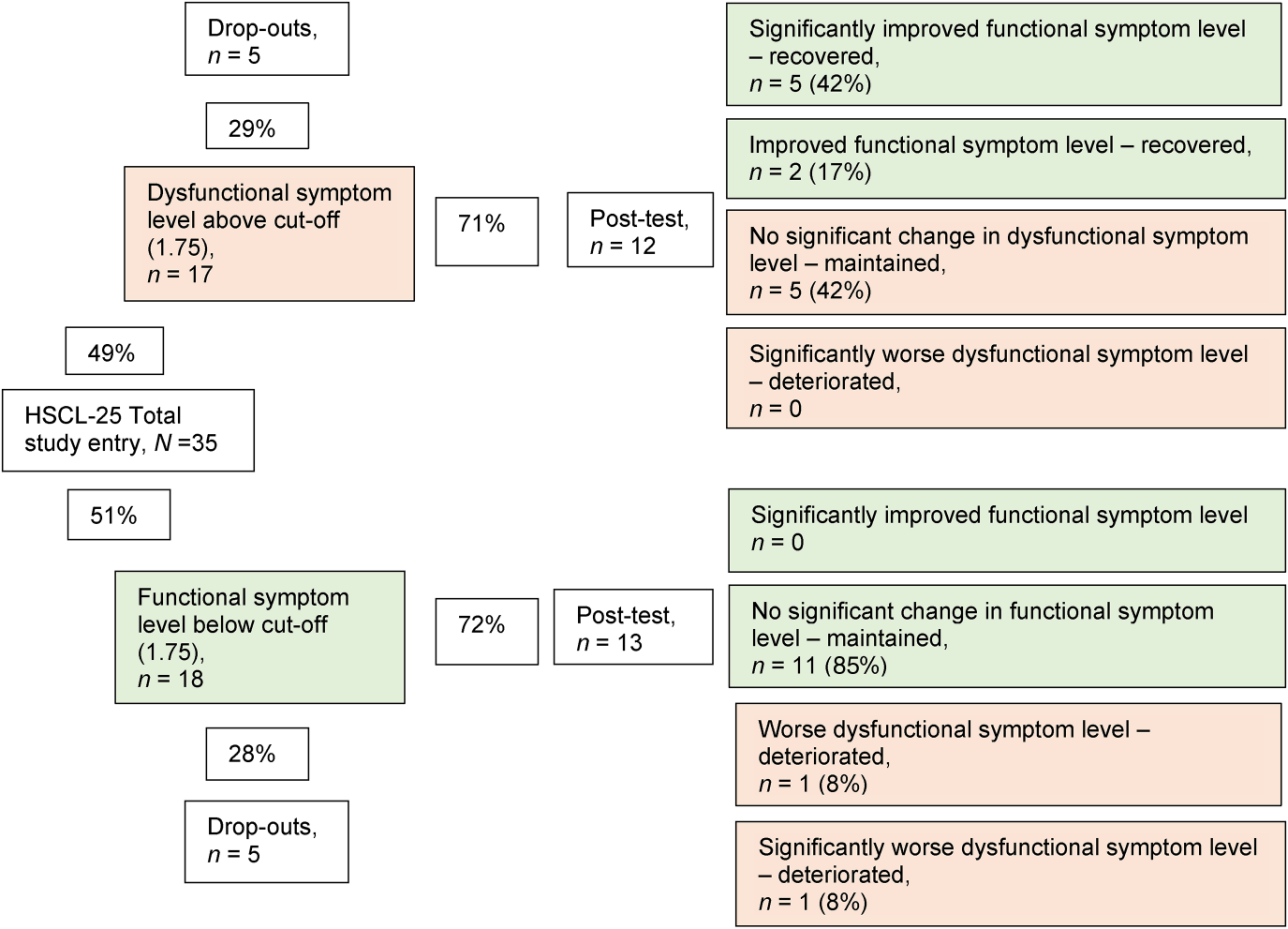
Categorizing individual-level change in global psychiatric symptoms (HSCL-25) using the Reliable Change Index (RCI).

*Note.* HSCL-25 = Hopkins Symptom Checklist-25 (Derogatis et al., 1974).

Almost half (*n* = 17 of 35) of the mothers reported dysfunctional levels of global psychiatric symptoms pretreatment. The change after intervention was calculated using RCI, and five mothers displayed significant improvements to functional levels. Among the mothers who did not have dysfunctional symptom levels before taking part in PS, one deteriorated significantly. Nineteen (76%) of the mothers experienced no significant changes in global psychiatric symptoms. Postintervention, over a quarter (28%, *n* = 7 of 25) of the mothers still had dysfunctional levels of global psychiatric symptoms (Figure 1).

#### Posttraumatic Stress Symptoms Assessed With IES-R

Mothers became less avoidant after taking part in PS (avoidance, IES-R), but the effect sizes were small (0.39) (Table 1).

**Figure 2.**
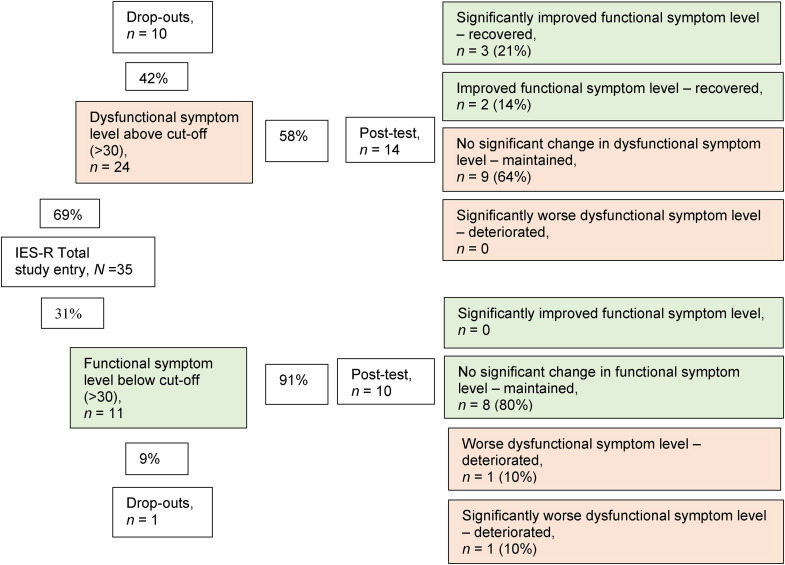
Categorizing individual-level change in trauma symptoms (IES-R) using the Reliable Change Index (RCI). *Note.* IES-R =* *Impact of Event Scale–Revised (Creamer et al., 2003).

About two-thirds (*n* = 24 of 35) of the mothers reported dysfunctional levels of trauma symptoms (IES-R) preintervention. The pre- to postintervention change was calculated using RCI: postintervention, 10 mothers with dysfunctional levels had dropped out, whereas three mothers had significantly improved and were considered recovered, as they scored below the cut-off limit for posttraumatic stress symptoms. Two mothers displayed increased symptoms postintervention, one having significantly deteriorated to dysfunctional levels of symptoms. Overall, for 20 (83%) of the mothers who had completed the intervention, there were no significant changes in posttraumatic stress symptoms. Of the 14 mothers who had dysfunctional levels preintervention, nine (64%) remained unchanged, and of the 10 mothers who were below the cut-off preintervention, two deteriorated to dysfunctional levels. In summary, postintervention with PS, almost half (46%, *n* = 11 of 24) of the completing mothers still had dysfunctional levels of trauma symptoms (Figure 2).

### IPV Exposure and Mothers’ Psychiatric Symptoms Preintervention

Higher levels of trauma symptoms (IES-R) were associated with high exposure to IPV (CTS2), regardless of its form. Mothers who suffered from high levels of the trauma symptom arousal (IES-R) and global psychiatric symptoms (HSCL-25) had high exposure to psychological violence (CTS2) (Table 2).

**Table 2.** Spearman Correlation (Two-Tailed) Between Maternal Scores Preintervention Concerning Psychiatric Symptoms (IES-R and HSCL-25) and Exposure to IPV (CTS2).

	IES-R intrusion	IES-R avoidance	IES-R arousal	IES-R total without arousal	IES-R total	HSCL-25
*CTS2 psychological*	.653**	.497**	.453**	.625**	.611**	.344*
*CTS2 physical*	.474**	.344*	.324^#^	.440**	.428**	.146
*CTS2 sexual*	.540**	.451**	.324^#^	.526**	.486**	.268
*CTS2 injury*	.584**	.505**	.301^#^	.589**	.526**	.164
*CTS2 total*	.641**	.473**	.454**	.609**	.598**	.328^#^

*Note.* CTS2 = Conflict Tactic Scale 2 (Straus et al., 1996); HSCL-25 = Hopkins Symptom Checklist-25 (Derogatis et al., 1974); IES-R =* *Impact of Event Scale–Revised (Creamer et al., 2003).

**Correlation is significant at .01 level (two-tailed).

*Correlation is significant at .05 level (two-tailed).

# Correlation is significant at .10 level (two-tailed).

#### IPV Exposure and Individual Global Psychiatric Symptoms (HSCL-25) Preintervention

Mothers with dysfunctional levels (above the cut-off limit) of psychiatric symptoms (HSCL-25) had been exposed to more psychological violence (*p =* .016, *Z* = –2.556) and total violence (CTS2) (*p =* .016, *Z* = –2.412) than had mothers who did not display dysfunctional symptom levels (Table 3).

**Table 3.** A Mann–Whitney *U* Test Was Used to Measure Mothers’ Exposure to IPV (CTS2) and Amount of Social/Emotional Support, Based on Levels (Above and Below Cut-Off Limit, Respectively) of Psychiatric Symptoms Measured with HSCL-25 and IES-R. The Table Presents Significant Pre- and Posttreatment Results.

CTS2	IES-R *Comparison of two groups: mothers with levels above and below trauma symptom cut-off limit, pre- and posttreatment*				HSCL-25 *Comparison of two groups: mothers with levels above and below global psychiatric symptom cut-off limit, pre- and posttreatment*					
	Sign, pretreat	*M* above cut-off	SD above cut-off	Sign, posttreat	Sign, pretreat	*M* above cut-off	SD above cut-off	Sign, posttreat	*M* above cut-off	SD above cut-off
		*M* below cut-off	SD below cut-off			*M* below cut-off	SD below cut-off		*M* below cut-off	SD below cut-off
Psychological	−2.18*	77.9	18.3	n.s.	−2.56*	89.4	65.4	n.s.		
		22.2	18.3			33.6	41.3			
Physical	n.s.			n.s.	n.s.			−1.66^#^	52.6	92.8
									3.3	6.6
Sexual	−1.87^#^	31.6	55.3	n.s.	n.s.			−2.08*	39.3	66.2
		0.5	1.3						14.1	40.1
Injury	−2.27*	12.1	25.9	n.s.	n.s.			n.s.		
		0.4	1.7							
Total	−2.19*	151.5	187.0	n.s.	−2.41*	175.9	201.5	n.s.		
		25.9	22.3			47.2	80.9			
Social and emotional support										
Frequency, sessions/month	n.s.			n.s.	n.s.			n.s.		
Time in session	−2.09*	1.9	0.3	n.s.	n.s.			n.s.		
		2.2	0.3							

*Note.* CTS2 = Conflict Tactic Scale 2 (Straus et al., 1996); HSCL-25 = Hopkins Symptom Checklist-25 (Derogatis et al., 1974); IES-R =* *Impact of Event Scale–Revised (Creamer et al., 2003).

**Significant at .01 level (two-tailed).

*Significant at .05 level (two-tailed).

# Significant at .10 level (two-tailed).

#### IPV Exposure and Individual Posttraumatic Stress Symptoms (IES-R) Preintervention

Mothers with dysfunctional levels of trauma symptoms (IES-R) had been exposed to more psychological violence (*p *= .030, *Z = *–2.175), injury (*p = *.023, *Z = *–2.270), and total violence (*p *= .029, *Z = *–2.190) than mothers who did not display dysfunctional symptom levels (Table 3)*.*

#### IPV Exposure and Individual Global Psychiatric Symptoms (HSCL-25) Postintervention

Mothers who had dysfunctional levels of psychiatric symptoms (HSCL-25) had been exposed to more sexual violence (*p = *.038, *Z* = –2.078) than mothers who did not display dysfunctional symptom levels (Table 3).

#### IPV Exposure and Individual Posttraumatic Stress Symptoms (IES-R) Postintervention

There were no differences in the magnitude of exposure to violence (CTS2) between mothers who were above versus below the cut-off limit for trauma symptoms (IES-R) postintervention (Table 3).

### Social and Emotional Support in the PS Intervention

The social and emotional support given to the mothers at each session ranged from 1.3 to 2.4 with a mean of 2.0, indicating that the PS sessions consisted of a mixture of both social and emotional support (26–75% of the session duration) and parenting skill practice in line with the PS manual. The mean duration of maternal participation in PS was 8.8 months (SD = 3.7, range 1.2–15.4 months), and mothers received a mean of 17.3 sessions (SD = 9.2, range 8–45 sessions) at a frequency of 2.1 sessions per month (SD = 1.0, range 0.8–5 sessions/month). Deviation from the weekly sessions recommended by the PS manual occurred when planned sessions were cancelled by either the mothers (due to sick children, own sickness, or need to work) or the counselors (due to sickness or sick children). The mean value for counselors’ satisfaction with their work assessed at each session was 7.23 (range mean 4.89–9.86, SD = 1.23), and the mean value of assessed maternal receptiveness was 7.53 (range mean 5.11–10, SD = 3.54).

For mothers as a group, the frequency and amount of time in sessions used for social and emotional support as assessed by the counselors were not associated with psychiatric symptoms (IES-R and HSCL-25) in the mothers (*p*_min_* *= .061, *p*_max_ *=* .831; *r*_min_ = –.380, *r*_max_ = .044) or with their exposure to IPV (CTS2) (*p*_min_ = .123, *p*_max_* =* .951; *r*_min_ = –.317, *r*_max_ = –.013).

Comparison of mothers as individuals, based on their levels of psychiatric symptoms (IES-R and HSCL-25) and clinical cut-off limits (functional or dysfunctional), indicates that mothers who had dysfunctional levels of trauma symptoms (IES-R total) preintervention received sessions that spent less time focusing on social and emotional support than did mothers with functional levels of symptoms. Frequency (i.e., number of received sessions per month) did not differ between mothers depending on their psychiatric symptom levels (IES-R or HSCL-25, functional or dysfunctional) or pre- to postintervention changes (Table 3).

When analyzing the results at the individual level, a difference could be noted between mothers living in domestic shelters and the other mothers. To explore this, we conducted an additional analysis.

### Mothers Living in Domestic Violence Shelters

Mothers living in domestic violence shelters (*n* = 9) had significantly higher levels of trauma symptoms (IES-R total, *M* = 52.39, SD = 12.93) than mothers who participated in PS as social service clients (IES-R total, *M* = 35.69, SD = 22.34, *p* = .034, *Z* = –2.114). The mothers living in the shelters also had higher exposures to IPV (CTS2 total, *M* = 181.89, SD = 172.57) than the other mothers (*M* = 87.83, SD = 159.90, *p* = .033, *Z* = –2.137).

Furthermore, mothers who lived in domestic violence shelters and completed the PS intervention (*n* = 6) had more frequent contact with counselors (*M* = 2.9 sessions/month, SD = 1.5), spent less time in treatment (*M* = 4.6 months, SD = 3.3, *p = *.004, *Z* = –2.856), and received less social and emotional support (*M* = 1.6, SD = .031, *p = *.004, *Z* = –2.861) than mothers who participated in PS as social service clients.

## Discussion

The general aim of this study was to explore the relationship between psychiatric symptoms, IPV exposure, and received social and emotional support in Swedish mothers who have participated in the PS intervention.

Mothers with the greatest exposure to IPV also showed the highest levels of psychiatric symptoms (HSCL-25 and IES-R). As a group, the mothers displayed decreased symptom levels after taking part in PS even if the sessions were not as frequent as the PS manual recommended. Counselors were generally satisfied with their work and reported that the mothers usefully assimilated and benefitted from the intervention. However, from an individual perspective, the symptom levels of most mothers remained unchanged, and less session time was spent on social and emotional support among those with the most severe psychiatric symptoms.

Swedish social services are obliged to offer support to mothers and children exposed to IPV, and feasibility studies of PS have shown that its positive results in terms of improved parental capacity and decreased behavioral disorders in children seem to be sustainable when the intervention is transferred from the US to Swedish contexts (Draxler et al., 2019, 2020). The empirical support for the effectiveness of PS, based on US and Swedish studies, supports dissemination of PS within Swedish social services. However, PS results from the USA have shown a weaker effect concerning improved maternal mental health, and this study conducted in a Swedish implementation setting seemingly replicates that finding.

Rauch et al. (2012) argued that mental health problems should be treated using interventions specifically targeting the specific mental health problem, such as trauma. The current findings, in conjunction with prior research on PS, substantiate the aforementioned hypothesis, suggesting that the inclusion of social and emotional support within the PS intervention did not appear to influence psychiatric symptoms in the mothers (Jouriles et al., 2009, 2010). Jouriles et al. (2009, 2010) discussed possible explanations, claiming that the emotional support offered in PS might not differ from regular support in treatment as usual and that PS might need to be improved or that additional interventions might be necessary. This also seems relevant to the needs of the mothers studied here, especially since they had higher pre- and postintervention levels of global psychiatric symptoms (summed HSCL-25 and GSI of 1.6 preintervention and 1.27 postintervention) than did the US sample (SCL90 and GSI of 0.67 and 0.56 preintervention and of 0.59–0.47 postintervention and follow-up) (Jouriles et al., 2009, 2010). When offering PS to IPV-exposed mothers with children who have developed behavioral disorders, Swedish social services should be aware and detect that some mothers need additional treatment for their psychiatric symptoms, especially posttraumatic symptoms. However, this poses a challenge as counselors within the Swedish social service are not trained to recognize psychological symptoms or to treat such symptoms.

The frequency of the sessions given to the mothers was lower than recommended, but this was not associated with their psychiatric symptoms (IES-R and HSCL-25) or exposure to IPV (CTS2). The amount of time the mothers received social and emotional support in the sessions was not associated with either psychiatric symptoms or exposure to IPV. The results indicated that the frequency and duration of social and emotional support given to the mothers were the same regardless of their levels of psychiatric symptoms or IPV exposure, with one exception: mothers living in domestic violence shelters while taking part in PS received less social and emotional support. The counselors were, however, generally very satisfied with their own work and believed that the mothers had received and responded positively to the intervention. It seems that there might be a gap between the counselors’ perception of maternal mental health and the woman's true needs. This might be because the counselors were administering PS for the first time and were so focused on the manualized treatment that they did not reflect on the mothers’ different needs. Another potential factor, in addition to the lack of training in identifying psychological symptoms, is the constrained accessibility of specialized mental health care in Sweden. Moreover, the recognition of this limited availability may further serve as an obstacle to acknowledging the mothers’ requirement for supplementary treatment.

In Swedish domestic violence shelters, the mothers often have someone to contact when needed, which might explain why mothers residing in shelters mainly received parenting skills training in PS and why their counselors spent less time offering social and emotional support. Perhaps the counselors implementing PS concentrated on practicing the parenting skills, while other shelter staff gave social and emotional support. It is quite possible that mothers living in shelters received not less but more such support, although not as part of the PS intervention.

In summary, mothers with high exposure to IPV tended to have higher levels of psychiatric symptoms. The social and emotional support given to the mothers was not associated with maternal symptoms or levels of IPV exposure. As this study lacked a control group, we do not know whether the observed effects are connected to PS or to other circumstances.

Notably, despite the mothers’ high levels of psychiatric symptoms, another study of the same sample found that they could improve their parenting capacity and that their children's behavioral problems decreased (Draxler et al., 2019). This supports the belief that mothers exposed to IPV could benefit from PS despite their mental health problems, managing to focus on their children and respond nonjudgmentally even in the context of IPV (Levendosky & Graham-Bermann, 2000).

For PS providers in Sweden, this highlights the importance of focusing on teaching parenting skills and also shows how difficult it might be to decide whether to continue with the intervention or to refer the mother to mental health care for additional/other treatment. Regarding feasibility, it is noteworthy that Swedish social service counselors are not educated in psychiatric evaluations, and our results indicate that counselors did not adapt or individualize the amount of social and emotional support to the mothers’ psychiatric symptoms or to the severity of IPV exposure. Swedish organizations providing PS would benefit from close collaboration with mental health care services so that counselors could receive support and supervision regarding whether and how psychiatric symptoms could interfere with the PS intervention. Furthermore, the social services need to arrange for the most vulnerable groups, such as mothers exposed to IPV, to receive regular weekly support. The current situation, in which counselors’ regulated work time is limited to office hours, whereas most mothers in Sweden work full or part time and do not receive financial compensation for participating in social service versus mental health care interventions, constitutes a possible obstacle to the mothers’ participation.

For mothers exposed to IPV and with psychiatric symptoms, a single intervention targeting mental health problems *or* parenting capacity seems insufficient, and in this study 28% (*n* = 7 of 25) and 46% (*n* = 11 of 24) of the mothers still had dysfunctional levels of global psychiatric and trauma symptoms, respectively, after intervention. This entails challenges for Swedish social workers, organizations, and research. Social workers require relevant knowledge in order to understand how IPV affects mothers’ mental health and parenting capacity. They also need to know when to refer the mothers to other and/or additional services. Organizations must be able to collaborate, support clinicians, and keep the family's best interest in focus.

This study also highlights the challenges of conducting research in the IPV field and in a naturalistic setting, especially when the studied mothers are still exposed to threats and violence. Many of the dropouts (*n* = 10) were mothers suffering from posttraumatic symptoms above the clinical cut-off, and most of them were living in shelters. This indicates that the most vulnerable families do not even obtain support or treatment. Hopefully, further research using different methods to target different aspects of IPV and their mental health consequences will give organizations and clinicians better knowledge of how to best support and reach families exposed to IPV.

### Strengths and Limitations

This study was a very first step in evaluating the implementation of PS outside the US, with a focus on the social and emotional support components. The mothers’ symptoms and exposure to IPV were studied at both group and individual levels, which improves the understanding of the intervention’s impact on clinical outcome. The study was performed in a naturalistic Swedish context of social service agencies, where counselors worked with PS as part of their regular work and resources. Participants were included in the intervention based on the agencies’ regular decision-making processes, as opposed to being specifically selected, which strengthens aspects of external validity. Regarding confounders and the threat of single operation and narrow stimulus sampling, these were ruled out as the treatment was given by 30 different counselors. However, the naturalistic context also implies difficulties, and the study has several limitations. Organizational barriers such as different decision-making processes (i.e., selection bias), possibilities to meet the mothers on a weekly basis, and employee turnover (i.e., method fidelity) are among the drawbacks of the naturalistic approach.

Since the study was a first try of PS, the sample was small, and the lack of a control group weakens the study's results and threatens the internal validity. The intervention was offered to Swedish-speaking mothers only, which excludes non-Swedish-speaking mothers, such as newly arrived refugees and immigrants. Further, results were based on completers, there were substantial numbers of attrition, and presumably, those included mothers that had sufficient resources/characteristics or experiences which could bias the results. This, as well as the lack of follow-up assessments of the mothers’ mental health, threatens the external validity. However, a follow-up assessment could not be conducted due to limited resources. Given the limitations inherent in this study, as well as the novelty of investigating the social and emotional support treatment component of PS, it is advised that the results are interpreted with appropriate caution and consideration.

Subsequent research should aim to address and thoroughly investigate the pivotal factors that ascertain the opportunities and willingness of IPV-exposed mothers to actively participate in supportive programs, such as PS. Furthermore, it is imperative for research endeavors to explore efficacious strategies that can effectively facilitate their engagement in both research studies and broader treatment contexts. To attain a more robust validation of previous findings, it is highly desirable to conduct a meticulously designed randomized controlled study, incorporating comprehensive follow-up assessments. Additionally, it is of paramount importance to examine the long-term organizational capacity of Swedish social services in implementing and sustaining the PS intervention over an extended duration.

## Conclusions

The present results indicate that the PS intervention was associated with a decrease in the trauma symptom of avoidance and in global psychiatric symptoms. However, from an individual clinical perspective, most of the mothers’ symptoms remained unchanged, a few mothers improved, and a few deteriorated. This indicates that some mothers needed additional treatment due to mental health problems that remained unchanged after PS treatment. Mothers with high exposure to IPV (CTS2), regardless of its form, had higher levels of trauma symptoms (IES-R), and the amount of social and emotional support was not individualized to their situation. This study highlights the need to consider multiple research aspects and methods as well as the importance of individual clinical assessments to address whether additional treatments are necessary.
